# Automated Recognition of RNA Structure Motifs by Their SHAPE Data Signatures

**DOI:** 10.3390/genes9060300

**Published:** 2018-06-14

**Authors:** Pierce Radecki, Mirko Ledda, Sharon Aviran

**Affiliations:** Biomedical Engineering Department and Genome Center, University of California Davis, Davis, CA 95616, USA; peradecki@ucdavis.edu (P.R.); maledda@ucdavis.edu (M.L.)

**Keywords:** RNA structure, HIV, RRE, structure probing, SHAPE, HMM-GMM, machine learning

## Abstract

High-throughput structure profiling (SP) experiments that provide information at nucleotide resolution are revolutionizing our ability to study RNA structures. Of particular interest are RNA elements whose underlying structures are necessary for their biological functions. We previously introduced *patteRNA*, an algorithm for rapidly mining SP data for patterns characteristic of such motifs. This work provided a proof-of-concept for the detection of motifs and the capability of distinguishing structures displaying pronounced conformational changes. Here, we describe several improvements and automation routines to *patteRNA*. We then consider more elaborate biological situations starting with the comparison or integration of results from searches for distinct motifs and across datasets. To facilitate such analyses, we characterize *patteRNA*’s outputs and describe a normalization framework that regularizes results. We then demonstrate that our algorithm successfully discerns between highly similar structural variants of the human immunodeficiency virus type 1 (HIV-1) Rev response element (RRE) and readily identifies its exact location in whole-genome structure profiles of HIV-1. This work highlights the breadth of information that can be gleaned from SP data and broadens the utility of data-driven methods as tools for the detection of novel RNA elements.

## 1. Introduction

RNA is one of the most important molecules for the formation, evolution, and regulation of life [1,2]. Although it is known that RNA serves important roles at nearly all levels of cellular function, the fundamental role of RNA in biological systems has remained constant: to encode genetic information, regulate genes and serve as a catalyst of biochemical reactions [1,3,4,5]. Within these contexts, the ability of RNAs to fold into specific structures is critical. For instance, the functions of thermosensors, riboswitches, aptamers, G-quadruplexes, and protein–RNA complexes all depend on the formation of intricate secondary and tertiary structures [6,7]. The continued discovery of such functional elements has necessitated the development of methods to obtain accurate structure predictions at high-resolution. To this end, X-ray crystallography and nuclear magnetic resonance are currently the ideal RNA structure characterization methods. However, their cost, labor requirements, and limited applicability render them low-throughput. More recently, structure profiling (SP) experiments have received considerable attention as an alternative approach for probing RNA structure that is more affordable and suitable for high-throughput applications. By providing a snapshot of the structural states of an RNA transcript at nucleotide resolution, SP experiments aim to elucidate the role of RNA structure in biologically relevant contexts [6,7,8,9].

Structure profiling experiments utilize chemical or enzymatic reagents that modify or cleave nucleotides in a structure-dependent manner. Modifying reagents are sensitive to the local stereochemistry of the RNA, meaning regions which are flexible are more likely to be accessible to the reagent. As a result, accessible regions are modified more frequently compared to regions that are rigid, internalized, or obstructed. Sites of modification lead to transcription terminations or to mutations, which are then detected by sequencing. The degree of modification, termed *reactivity*, is then quantified, providing nucleotide-resolution information on a transcript’s structure. The sequence of reactivities over a transcript is termed a *structure profile*. Structure profiling experiments were recently scaled to transcriptome-wide levels with the advent of next-generation sequencing. These advances have revolutionized our ability to study RNA structure at the scale of the entire transcriptome and in the complex context of a living cell, with new applications and methods continuing to emerge [7,9,10,11].

Despite the recent breadth and scale of SP datasets, universal and efficient tools for their interpretation and analysis are generally lacking. There are several reasons for this, one being the difficulty in integrating nucleotide-resolution measurements to the level of biologically relevant structural elements [12]. This is critical because RNA function is typically driven by structural elements that span at least a few and often tens of nucleotides. Examples of functional elements with available consensus structures that are impacted by cellular conditions include aptamers and riboswitches, which respond to ligands [13,14,15,16,17], thermosensors that respond to temperature [18], G-quadruplexes [19,20,21,22], as well as several non-coding RNAs [23,24]. Additionally, RNA modifications, which are prevalent and dynamic, can modulate structures [25,26]. Traditional approaches to study such elements often rely on secondary structure prediction via thermodynamic models and dynamic programming algorithms, fused with SP data [27,28,29,30,31]. While powerful, these methods do not scale well to transcriptome-level analyses [32] and are often inaccurate for long RNAs [33]. More importantly, they are based on modelling assumptions that fail to capture the full complexity of the cellular environment [34], in particular inter-molecular interactions and varying cellular conditions. In addition, RNA structures are dynamic as illustrated by cotranscriptional folding pathways [35,36]. In these contexts, it is valuable to be able to rapidly glean structural information from SP data alone. However, the diversity of available reagents, signal enrichment strategies, modification detection methods, and analysis pipelines results in disparate statistical properties of SP datasets. Consequently, existing SP-based methods are often specialized to the properties of the data at hand and to the study’s biological objectives [8,9,37,38,39].

To address these needs, we previously developed *patteRNA*, a machine learning algorithm for mining RNA structures from SP data directly [32]. Leveraging a simplified representation of RNA structures as chains of paired and unpaired nucleotides, *patteRNA* learns the statistical properties of two components that are fundamental to all SP datasets. The first is RNA structure. Here, *patteRNA* learns how paired and unpaired nucleotides come together to form commonly observed structural motifs, such as hairpins. This is accomplished by training a Hidden Markov Model (HMM) to capture the probability of adjacent nucleotides transitioning between paired and unpaired states, and vice versa. The second feature is the SP signal, irrespective of the SP strategy employed. In this context, *patteRNA* learns which reactivity values are expected for paired nucleotides and which values are expected for unpaired ones [40,41]. These expectations are formulated in a Gaussian Mixture Model (GMM) of reactivity values. When fused together, these two features give rise to a GMM-HMM framework [42], which allows *patteRNA* to bridge between the resolution of reactivity measurements (i.e., single nucleotide) and that of the sought-after structural elements (i.e., reactivity patterns over local regions). To implement this, the GMM-HMM statistically links every structure to every possible data pattern to assess their consistency. Equipped with these statistical modeling capabilities, *patteRNA* rapidly scans local data patterns in massive datasets, in search of regions where the data indicates that a target motif is likely to occur.

While we demonstrated *patteRNA*’s utility as a tool for automated mining of patterns in SP data, our previous work focused on detecting highly pronounced structural changes, and results derived from several datasets were generally considered in isolation [32]. To improve the algorithm’s robustness and ease-of-use, we present updates to its training routine, which is now fully automated. To extend the repertoire of *patteRNA*’s applications, we introduce improvements to its scoring pipeline, which now utilizes a normalization strategy to facilitate integration and direct comparison of search results conducted with different target motifs and datasets. Using the revised pipeline, we demonstrate our algorithm’s refined capabilities of pattern recognition. Specifically, using the human immunodeficiency virus type 1 (HIV-1) Rev response element (RRE) as an example, we show that *patteRNA* can discriminate highly similar structure profiles, identify the precise location of RRE with high confidence in a whole-genome profile, and capture changes to ensemble composition in simulated data. Overall, our results suggest that data-driven models are a promising route for the discovery of functional RNA elements. Our findings also serve as further validation of *patteRNA* and its capabilities as an automated and broadly applicable RNA structure mining engine.

## 2. Materials and Methods

### 2.1. Overview of Structure Profiling Experiments

Structure profiling experiments aim at querying all RNA structures in a sample at nucleotide resolution. Chemical reagents or enzymes are used to modify the RNA in a structure-dependent manner, i.e., flexible or unpaired nucleotides are more accessible to the chemical/enzyme and are modified more frequently [27]. A common approach using chemical reagents is SHAPE (selective 2${}^{\prime }$-hydroxyl acylation analyzed by primer extension), where modifications involve the formation of chemical adducts on hydroxyl residues of the RNA backbone. Commonly used SHAPE reagents include 1-methyl-6-nitroisatoic anhydride (1M6), 1-methyl-7-nitroisatoic anhydride (1M7), *N*-methylisatoic anhydride (NMIA), and 2-methylnicotinic acid imidazolide (NAI) [43,44]. Chemical adducts interfere with reverse transcription, leading to either complementary DNA (cDNA) transcription terminations or mutations, which are then read out by DNA sequencing. Using two experimental conditions, one with the reagent (treated sample) and one without it (control sample), one can infer from sequencing reads a rate of modification, called reactivity, at each nucleotide [45,46,47,48,49,50,51]. High and low reactivities are generally indicative of unpaired (less constrained) and paired (more constrained) nucleotides, respectively. Consequently, a structure profile correlates with the underlying assayed secondary structure.

### 2.2. Improvements to patteRNA’s Training Routine

#### 2.2.1. Building the Training Set Using Kullback–Leibler Divergence

To minimize the size of the training set, we start by compiling a histogram of all observed reactivities. The binning interval is determined automatically using the *auto* mode in the *histogram* function from the Python package *numpy* [52]. Next, transcripts are sorted in descending order of their data density, i.e., the proportion of observed values that are neither zero nor missing. Then, to build the training set, we sequentially add transcripts to our training set until its properties capture the distribution of the entire dataset. This agreement is quantified using Kullback–Leibler (KL) divergence [53] between the histogram of the entire dataset (P) and the one from the training set (Q). Note that both histograms are built using the same binning intervals to obtain probability density vectors of identical size. Formally, KL divergence ($D_{KL}\left(P||Q\right)$) is defined as:

(1) $$D_{KL}\left(P||Q\right)=\sum_{\forall i}P_{i}\log\frac{P_{i}}{Q_{i}}.$$

Transcripts are added until $D_{KL}\left(P||Q\right)$ becomes smaller than a pre-set criterion, by default 0.01. Note that a drastic reduction in training runtime is expected as the computational overhead associated with the computation of the KL-divergence is eclipsed by the training phase completing significantly faster when using a subset of the data instead of the full dataset.

#### 2.2.2. Determining an Optimal Number of Gaussian Components

To determine an optimal number of Gaussian components (*K*) per pairing state, we start by training the model with a single Gaussian per state (K=1). We then compute the model’s Bayesian Information Criterion (BIC), based on the number data points (*n*), the number of free parameters (ν) and the log-likelihood (logL) of the model, which is defined as:

(2) BIC=−2logL+νlogn.

Note that ν, the number of free parameters, is essentially an indicator of the model’s “complexity”. The BIC summarizes a model’s performance penalized for its complexity (the νlogn term) into a single metric and is commonly used in model selection [54]. The same procedure is then repeated with K+1 components until an increase in BIC is observed. Such increase indicates that the currently tested model is less appropriate than the previous, simpler, model and therefore an optimal *K* was found. The trained model derived from this *K* is then utilized for scoring.

#### 2.2.3. Parameter Initialization

Parameters can be initialized either in a supervised or unsupervised manner. For supervised initialization, we use known reference structures to compute both the HMM and GMM parameters deriving from them. Specifically, for the HMM, we set the initial and transition probabilities for each pairing state equal to the frequencies observed in the reference structures. For the GMM, we start by partitioning reactivities based on the known pairing states of the reference structures, resulting in two data distributions, one for paired and one for unpaired nucleotides [40,41]. We then fit a standard GMM, as implemented in the Python package *scikit-learn* [55], with a single Gaussian component (κ=1) to each state-specific distribution. Next, the BIC is computed for each fitted distribution and summed into a single metric describing the performance of the fit for the two pairing states. We then increment κ by 1, repeat this procedure, and stop when the summed BIC increases. Once an optimal κ is found, we use the resulting means, variances and weights for each component and pairing state as initial parameters.

For unsupervised initialization, the default initial parameters are listed in the Appendix A. Note that both means and variances depend on the input dataset. Specifically, under the initial assumption that the proportion of paired and unpaired nucleotides are identical, we can space Gaussians evenly across the data distribution using the percentiles of the reactivities distribution as shown in Figure S1. For variances, we initialize them as the variance of the entire data distribution.

### 2.3. Computing Raw patteRNA Scores

Using a trained model, *patteRNA* rapidly scores sites in the data for consistency with a target motif. Scoring consists of quantifying the nucleotide-wise agreement between the target motif and the considered site, using a probabilistic framework [32]. At each nucleotide in a scored site, we compute the probability ratio of the target path, *T*, over the inverse-target path, $T^{\prime }$. The inverse-target path is simply the opposite state sequence of the target. Because we only consider two pairing states (paired and unpaired), there exists only a unique $T^{\prime }$ for any given *T*. The probability ratio is derived from the GMM-HMM with the GMM capturing the likelihood of the target path given emission probabilities of reactivity values in the scored site, while the HMM captures the likelihood of the target given its state sequence as transition probabilities. It is subsequently log-transformed to handle nucleotides in the data where one pairing state is highly preferred over the other. At these nucleotides, the probability ratio would otherwise explode or collapse to exceedingly large or small values, leading to numerical overflow. The sum of log probability ratios is computed over nucleotides in the target site to produce a total raw score. More formally, we define a raw score as:

(3) $$\mathrm{score}\left(\mathrm{target}=T,\mathrm{site}=S,\mathrm{model}=\theta \right)=\sum_{\forall i}\log\frac{\mathrm{Prob}(S_{i}=T_{i}|\theta )}{\mathrm{Prob}(S_{i}=T_{i}^{\prime }|\theta )}$$

In practice, posterior probabilities at each nucleotide and for each pairing state are computed during training, hence scoring is a rapid process that simply involves log-transformation and summation of pre-computed values.

### 2.4. Sequence-Based Constraints

An important consideration when using *patteRNA* is the option to use sequence-based constraints. Simply put, sequence constraints are a set of rules describing which pairs of nucleotides are allowed to form base pairs. We follow the canonical set of valid base pairs when enforcing sequence constraints. Base pairs considered valid are G–C and A–U (Watson–Crick), as well as G–U (wobble). Note that, when enforcing sequence constraints, we do not output scores at sites whose sequence violates the constraints implied by the target structure. Visual examples of sequence-structure comparisons that pass or violate sequence constraints are summarized in Figure S2.

### 2.5. Comparative Motif Scoring

*patteRNA* normalizes raw scores by comparing them to the distribution of raw scores under the null hypothesis ($H_{0}$, defined as sites that do not harbor the target). To build the null distribution, we randomly sample raw scores from sites violating sequence constraints (see Section 2.4 in Materials and Methods). To do so, we scan all transcript sequences in a rolling window of the same length as the target path to create a pool of regions, from which we sample up to 5000 null raw scores (or as many as possible, if fewer than 5000 sites in the data violate sequence constraints). If sequence constraints are not enforced, we sample up to 5000 raw scores across the entire dataset.

Once null scores are compiled, we fit null distributions for each target motif using a skew-logistic (also known as a generalized logistic) probability density function (PDF). Optimal parameters are determined by maximum likelihood estimation using the implementation in SciPy [52]. We then normalize each raw score with respect to the target motif’s null distribution by determining the probability of observing a raw score greater or equal to it, also known as the survival function. This probability is log-transformed to output a *c*-score, which we write:

*c*-score = −log_10_(1 − *F*(score; *α*, *β*, *γ*)), (4)

where F(score) is the cumulative PDF of the fitted null for a target motif and {α,β,γ}, the shape, shift (location) and scale parameters, respectively. By definition, *c*-scores are always positive and not upper-bounded. Higher *c*-scores indicate that the considered site is more likely to harbor the target motif. Importantly, the log-transformation serves to convert the 1−F(score;α,β,γ) term, which is diminishingly small for sites likely harboring the target motif, to an easily interpreted normalized score. Null distributions with fewer than 100 samples are discarded, and normalized scores for the associated target motifs are not produced. In such cases, *patteRNA* outputs a warning to the user indicating the normalized scores are not computed because the null distribution cannot be estimated reliably.

### 2.6. Benchmarking patteRNA Scores

**Figure 1:**
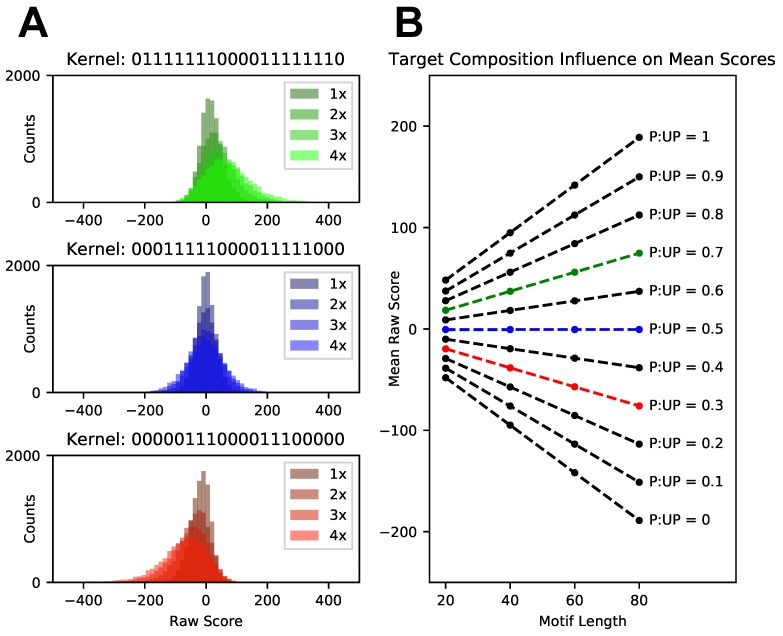
Distributions of raw scores associated with three target motif kernels. (**A**) Raw score distributions for three motif kernels of equal length and composed of 70% paired (top), balanced (middle), and 30% paired nucleotides (bottom). Overlaid are distributions for longer motifs obtained by concatenating kernels two (2×), three (3×), and four times (4×). (**B**) Mean raw scores for each of the distributions (red, blue, and green). The *x*-axis represents motif lengths corresponding to kernels repeated 1× (20-nt), 2× (40-nt), 3× (60-nt) and 4× (80-nt). Mean raw scores for distributions obtained when searching the Weeks set for additional kernels spanning a range of possible pairing state compositions are denoted in black. Dashed lines indicate a linear regression of the mean scores observed when extending a kernel’s length.

**Figure 2:**
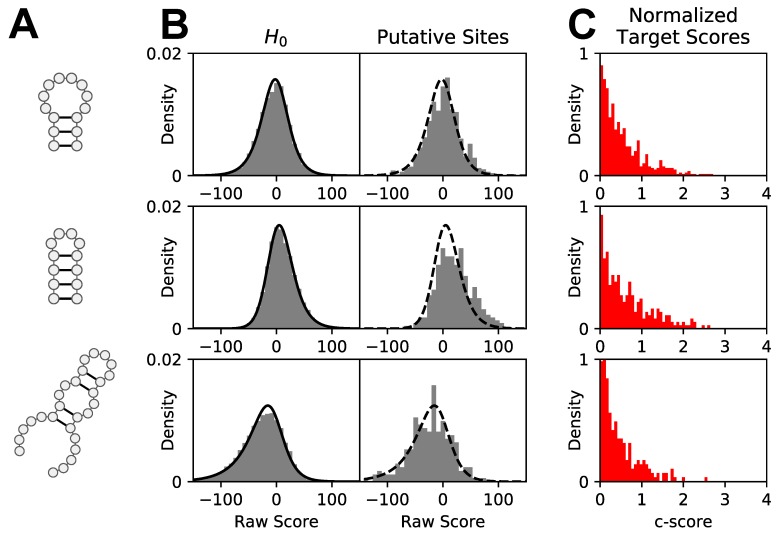
Normalization of *patteRNA* raw scores to *c*-scores. (**A**) Secondary structure of the target motifs. (**B**) Raw scores at null sites ($H_{0}$, left) and raw scores at putative sites satisfying sequence constraints (right). Null sites refer to sites where the RNA sequence precludes formation of the target motif. The solid black curves correspond to a skew-logistic density function fitted on the null scores. On the right panels, the same fitted density is superimposed (dashed curve) and is used to normalize target scores. (**C**) Distributions of normalized putative scores, i.e., *c*-scores.

To benchmark *patteRNA*’s normalization procedure against real data, we compiled a collection of 21 reference RNAs, referred to as the Weeks set [32]. This dataset was used to produce Figure 1 and Figure 2. In Figure 1, we scored the Weeks set for three target motif kernels: (1) 70% paired (state path: 01111111000011111110); (2) 50% paired (state path: 00011111000011111000); and (3) 30% paired (state path: 00000111000011100000). To investigate the effects of motif length, scores were also generated for each kernel when repeated two, three, and four times, as indicated by the 2×, 3×, and 4× labels in Figure 1A. Post-processing, statistical analysis, and figure generation were performed using in-house Python scripts. Training on the Weeks set used log-transformed reactivities and completed in 13 EM-iterations and in 5 s. Scoring of all benchmarking motifs was completed in 29 s.

To demonstrate our normalization pipeline, as shown in Figure 2, the following three target motifs were used: (1) hairpin, stem length 3 and loop length 8 (dot-bracket: (((........)))); (2) hairpin, stem length 4 and loop length 4 (dot-bracket: ((((....))))); and (3) hairpin-internal loop composite (dot-bracket: .....((..((.....))..)).....). Targets were scored using the same trained model obtained with the Weeks set, as described above in this section. Scoring and normalization to *c*-scores were completed in 8 s.

### 2.7. HIV Rev Response Element Mutant Analysis

Previous work by Sherpa et al. [56] on the structure of the RRE in HIV-1 resulted in SHAPE profiles for seven variants of RRE. Collectively, these seven SHAPE profiles are referred to here as the Sherpa set. Two of these variants correspond to isolated isomers of RRE separated via native polyacrylamide gel electrophoresis (PAGE); they are denoted 5SL (five stem-loop) isomer and 4SL (four stem-loop) isomer. The other five profiles were generated from five RRE mutants (Mutants A–E) designed to stabilize or disrupt the two native forms. The seven RRE SHAPE profiles in the Sherpa set, each 232 nucleotides in length, were used collectively to train *patteRNA*. It is noted that the predicted structures of 5SL and 4SL are identical to Mutants A and B, respectively, hence the Sherpa set is comprised by seven SHAPE profiles with six unique nucleotide sequences predicted to give rise to five unique secondary structures. The full RRE structures are shown in Figure S3. *patteRNA* was then used to score the seven profiles for both their full-length predicted structures (232-nt) and the SL III/IV region (59-nt). Thus, each profile received five full-length scores as well as five scores at each possible 59-nt window, or a total of 5+5×(232−59)=1326 scores. Data were log-transformed prior to *patteRNA*’s run, hence the --log argument was not used. Analysis was performed twice, with and without sequence constraints enforced. Training converged in 61 iterations and 30 s. Scoring and normalization was completed in 3 s.

### 2.8. Searching the HIV Genome for Rev Response Element Motifs

**Figure 3:**
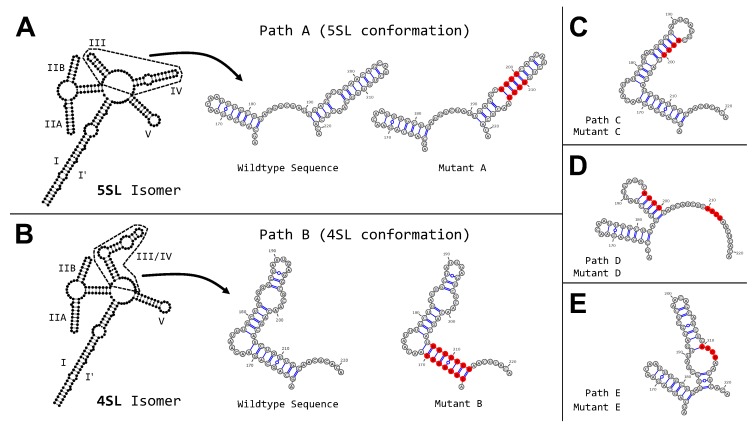
Predicted secondary structure of the Rev response element (RRE). (**A**) Full-length predicted structure of the five stem-loop (5SL) isomer of RRE. Stem-loops are indicated by their numeral. The region of interest (SL III/SL IV) is indicated with dashed lines, and expanded to show base pairings. The structure of RRE Mutant A, a variant of RRE that prefers the 5SL conformation, is also shown. (**B**) Full-length predicted structure of the four stem-loop (4SL) isomer of RRE along with a similar comparison as made in (**A**). Shown to the right is the structure of Mutant B, an RRE variant preferring the 4SL conformation. (**C**–**E**) Predicted secondary structure of the SL III/IV region for three additional RRE mutants. All mutants were produced by Sherpa et al. [56] with induced mutations highlighted in red.

RRE motifs were searched in four whole-genome structure profiles of HIV-1, three of which were generated by Siegfried et al., who employed high-throughput mutational profiling in conjunction with 1M7, 1M6, and NMIA SHAPE reagents (SHAPE-MaP) [47]. The fourth profile, generated by Watts et al. [57], was obtained with the 1M7 SHAPE reagent and capillary-based cDNA quantification. *patteRNA* was trained on each profile independently using log-transformed reactivities. The trained model for each HIV genome was subsequently used to score sites in the data for similarity to all five full-length structures of RRE from the Sherpa set as described in HIV RRE Mutant Analysis (see Figure 3). When scoring, sequence constraints were not enforced, thereby generating five scores for every possible 232-nt window. Sequence constraints were not enforced because we sought to assess how scores compared between the known site of RRE and other sites in the genome that violate sequence constraints. Training converged in under 100 iterations and 3 min for all profiles. Scoring was completed for all profiles in under 90 s, for a total runtime per genome of approximately 2–4 min.

Each profile was then scored for the presence of the 59-nt SL III/SL IV region as represented in Figure S4. Scoring was performed with and without sequence constraints. With sequence constraints, the search space was consequently reduced to only the exact location of SL III/SL IV in the genome (nt 7409–7467) (i.e., no other sites in the genome satisfied sequence constraints). Furthermore, only Paths A, B, and E satisfied the sequence constraints at this site, so only scores from these paths are reported. Without sequence constraints, scores were generated at every possible 59-nt window within the HIV-1 genome. Using the associated trained model, scoring was completed for each profile in under 30 s.

To compare *c*-scores directly between searches in the HIV-1 genome and a larger dataset, we utilized publicly available in vitro transcriptome-wide PARS data (reference GM12878) from Wan et al. [37]. The data were processed as described previously, and the same trained model was used [32]. Using the revised pipeline, we scored the full-length 5SL and 4SL RRE conformations at 1,114,957 possible sites on 649 transcripts with at least 75% data density (i.e., ≤25% missing values) from the PARS dataset. Searches were conducted without sequence constraints and scoring was completed in about 8 min. We then ranked *c*-scores obtained at the location of the RRE in all HIV-1 SHAPE profiles from the Siegfried and Watts sets directly against *c*-scores obtained with searches in the PARS data.

### 2.9. *In Silico* SHAPE Mixtures of HIV-1 Structure Variants

SHAPE profiles were created in silico to emulate mixtures of pure 4SL and 5SL conformations, as isolated by Sherpa et al. Synthetic mixture profiles were created in 10% increments from 100% 5SL to 100% 4SL by taking a weighted average of the 4SL and 5SL reactivities at each nucleotide. Each mixture was then scored against the 5SL and 4SL 59-nt target paths of the SL III/SL IV region (see Figure 3 and Figure S4), using a model trained from the seven profiles in the Sherpa set.

## 3. Results

### 3.1. Overview of patteRNA Workflow

*patteRNA* first reads a dataset to train its HMM-GMM model. After training, the model can be used to mine for user-specified structures (referred to as target motifs). During this phase, which we call *scoring*, *patteRNA* attributes a score to each considered region in the input RNAs, which we call a *site*. The score is computed as the log ratio of the probability of the target motif over the probability of the target motif’s inverse (see Section 2.3 in Materials and Methods). A higher score indicates that a site is more likely to harbor the target motif. Central to our method is a simplified representation of secondary structures (target motifs) as a sequence of nucleotides in one of two pairing states, namely, paired (denoted by 1) or unpaired (denoted by 0). We hereby use the term *path* to refer to a sequence of consecutive nucleotide pairing states as represented in *patteRNA*. Note that this is a simplification of the conventional representation of secondary structures, where the requirement to specify pairing partners is eliminated, as these are not revealed by SP data.

### 3.2. Score Normalization for Comparative and Integrative Analyses

When scoring a dataset against a single target motif, it is straightforward to parse which scores correspond to sites where the motif is more likely to occur: simply rank sites by their scores and look for top-scoring ones. However, when scoring a dataset against multiple target motifs and collectively considering the results of these searches, rank-based analysis is insufficient. At the root of this issue is our observation that scores can be biased due to properties of the target motif. Each target motif produces a distribution of scores that might vary greatly in its statistical properties and dynamic range. Such discrepancies pose a challenge to both integrative and comparative analyses of *patteRNA*’s outputs, as they render scores incomparable between distinct searches. This is particularly relevant when conducting searches for functional elements that can fold into several plausible conformations, when comparing a motif and its sub-motifs components, or for comparative analysis across varying experimental conditions [12,58,59,60,61]. For example, if scores for motif A span a different range than scores for motif B, a rank-based analysis of scores between A and B is not appropriate as these scores originate from different distributions. To illustrate this point, consider scores for three 20-nt motifs with paths “01111111000011111110” (70% of nucleotides are paired), “00011111000011111000” (50% paired), and “00000111000011100000” (30% paired). Figure 1A shows raw score distributions for these target motifs when searching across a reference set of 21 in vitro SHAPE profiles, which we call the Weeks set [32,41,62,63,64]. Score distributions when two (2×), three (3×), and four (4×) repeats of the state-sequences for these motifs are concatenated and searched are also included in these plots to assess the effects of a target motif’s length, without affecting state composition (i.e., the proportion of paired to unpaired states). In this context, the original 20-nt paths (1×) are denoted as the “kernels” of the concatenated forms (2×, 3×, and 4×). Immediately apparent is a drastic difference in the mean and skew of score distributions associated with each motif in Figure 1A. There are two main issues with this. First, one cannot merge and then rank scores from multiple searches to infer which sites are likely to harbor any of the sought targets, as certain searches might dominate the top of the list. This, in turn, warrants separate analysis of each search. Second, scoring a site of interest against two alternative targets might not reveal which target is more likely to be present.

The statistical properties of score distributions were found to primarily depend on the length of the target, its state composition, and the proportion of predicted paired/unpaired nucleotides in the data. Firstly, longer targets generally give rise to score distributions with larger variances. This is because scores are constructed as a sum of log ratios of probabilities at each nucleotide in the scored region (see Equation (3) in Materials and Methods) [32]. Consequently, scores for longer targets involve summation over a larger number of terms, each with their own variance, thereby leading to overall increased spread. This bias can be seen in Figure 1A, where distributions of scores expand as progressively longer motifs are scored. Secondly, shifts to the mean of a score distribution are driven by an imbalance in the state composition (i.e., paired/unpaired ratio) of the target motif. To illustrate this point, the means of the score distributions for the target motifs described earlier are shown in Figure 1B, where each kernel (green, blue, and red) has a unique composition. Results show that means are influenced by the target’s length (*x*-axis) and state composition (individual curves). Note that we also observed that the magnitude of the shift in the mean is proportional to the composition of predicted pairing states across all nucleotides in the data (data not shown). Additionally, although the state composition of the target and the state composition of the data both influence the mean, we found that if the state composition of the target is balanced (i.e., 50/50 paired/unpaired), the mean of the distribution will be at zero regardless of the imbalance in the data.

To allow meaningful comparisons of *patteRNA* scores across datasets and target motifs, we developed a normalization strategy that, given a target, accounts for the statistical properties of its scores in a given dataset. The normalization step results in a comparative score, termed *c*-score, which quantifies the statistical significance of a site’s raw score, given an estimated null distribution of raw scores associated with the target. Hereafter, the term “raw score” refers to *patteRNA* scores as described previously [32], while the term *c*-score refers to normalized scores. To determine the significance of a raw score, we require a distribution of raw scores (null distribution) at sites that do not harbor the motif (our null hypothesis, $H_{0}$). In practice, we do not know with absolute certainty where a motif will not occur. However, by using nucleotide sequence information, we can identify sites that are highly unlikely to harbor a motif because non-canonical base pairings would be required to give rise to the target motif. Specifically, sites where the nucleotide sequence allows for the formation of the motif via Watson–Crick or wobble base pairs are considered as putative positives. Conversely, sites that preclude motif formation are classified as falling under the null hypothesis. This filtering process is hereby called “sequence-based constraints.” By applying sequence constraints and randomly sampling null sites, we can approximate the score distribution under the null hypothesis. Given the null distribution, a *c*-score for a given raw score, *r*, is the −$\log_{10}$ of the probability of observing raw scores that exceed *r* (in other words, the area under the null distribution above *r*). Note that logarithmic transformation is applied to increase the separation between diminishingly small values which are strongly indicative of the presence of the target motif, similarly to common practices in genome-wide association studies [65]. As such, *c*-scores are always positive and not upper bounded, and a larger *c*-score is indicative of a stronger match between targets and scored sites. The null distribution is then fitted using a skew-logistic PDF. The rationale for a parametric description of the null is that it allows inferences in situations where the considered raw score falls outside the range of the null raw scores. The choice of a skew-logistic PDF was motivated by our observation that null distributions are generally non-Gaussian and often skewed (see Figure 1A). Note that, if sequence constraints are not enforced, the null distribution is instead constructed using scores from all sites in the data (see Materials and Methods). Under these circumstances, the null distribution will be biased, as it includes scores from true positive sites. Nevertheless, if the target motif is not widespread in the data, which is commonly the case, then this bias will only marginally affect *c*-scores as the null distribution will still contain a vast majority of negative sites.

An illustration of how our normalization framework converts raw scores at putative positive sites to final *c*-scores is shown in Figure 2. We illustrate the normalization process for three target motifs, namely, a short stem/long loop hairpin, a long stem/short loop hairpin and a hairpin-internal loop composite (Figure 2A). First, *patteRNA* computes raw scores at sites precluding formation of the target motif. These scores are used to approximate the true distribution of scores under the null ($H_{0}$) hypothesis (Figure 2B, left panels). A skew-logistic density function is then fitted to these null scores (black curve) and used to quantify significance of raw scores at putative sites (Figure 2B, right panels, where the overlaid dashed curve is the fitted null distribution). Finally, raw scores at target sites are converted into *c*-scores (Figure 2C) using the null distribution.

Differences in null distributions, distributions of raw scores from putative sites, and *c*-score distributions convey important observations from our normalization pipeline. First, null distributions for the three target motifs in Figure 2A differ in their statistical properties, and the skew-logistic PDF models observed data with high fidelity. Secondly, the distribution of raw scores from putative sites are different compared to their associated nulls. Namely, putative scores from a 4-nt stem/loop hairpin (Figure 2, middle) are noticeably shifted toward positive values compared to the null. In line with expectations, this shows that sites satisfying sequence constraints are more likely to emit SHAPE data in agreement with the presence of that hairpin. Although a similar shift exists for the hairpin with a shorter stem and a longer loop (Figure 2, top), the distinction is less dramatic. We presume that this is due to the longer loop destabilizing the hairpin more frequently compared to a hairpin with a shorter loop, which is generally assumed to be more stable. In addition, this can also be driven by sequence constraints not filtering out sites where a hairpin with a shorter loop would be feasible. In other words, while we considered a hairpin with a long loop at a site, it is more likely that the site harbors a hairpin with a shorter loop and a longer stem if the sequence permits it, as this would be energetically favorable. The distribution of scores at putative sites for the third motif, a short hairpin containing an internal loop, closely follows the null distribution, suggesting that this target is not commonly present in the data and sequence constraints alone are a weak indicator of the motif’s presence. Finally, the distribution of *c*-scores reflect these relative differences. Namely, there is an enrichment of *c*-scores greater than 1 for the short-loop hairpin that is more pronounced compared to the long-loop hairpin (Figure 2C, top and center panels). Comparatively, this enrichment is absent for the third motif (Figure 2, bottom panel).

In summary, we have demonstrated that *patteRNA*’s raw scoring scheme is subject to biases arising from a target motif’s paired/unpaired composition as well as its length. Moreover, we observed an additional bias due to the proportion of paired/unpaired nucleotides in the dataset. As we highlighted, these biases preclude a direct comparative analysis of different target motifs across datasets. To improve our algorithm’s ability to assess relative significance of target scores, we developed a normalization pipeline that produces *c*-scores, which provide a more meaningful metric with which to interpret results from distinct searches.

### 3.3. Targeted Search of Alternative Motifs in HIV-1

Essential to viral replication and RNA trafficking in HIV is the Rev-RRE regulatory system [66]. The RRE is an RNA element present in all unspliced and partially spliced viral mRNA transcripts from an HIV-infected host cell [67]. The viral protein Rev localizes to the nucleus and binds to RRE in a cooperative manner, forming the Rev-RRE complex. Next, Crm1 and other host proteins are recruited by the Rev-RRE complex, which is then exported to the cytoplasm along with its attached mRNA transcript. Due to its highly-structured nature and implications in HIV replication, RRE has been subject to extensive structural analysis. Its structure has been characterized by crystallography [68,69], small-angle X-ray scattering [70,71], probing experiments [47,57,71,72,73,74], and other methods [75,76]. Even with the wealth of data collected, the secondary structure of RRE has remained controversial. Studies have arrived at either a 4SL [73,74,77] or a 5SL [57,72,75] structure, although slightly deviant structures have also been suggested [71]. The two principal structures, 4SL and 5SL, are shown in Figure 3A,B. These competing conformations are largely identical. Both predict the formation of a central loop, from which a number of stem-loops fold. The specific region of RRE that has remained controversial is the SL III/SL IV region (nt 163-221, see dashed frames in Figure 3A,B). It is believed that SL III and SL IV either exist as two separate stem-loops (5SL structure) or combine to form a larger stem-loop, denoted SL III/IV (4SL structure). Notably, although the mesoscale structural arrangements of these two conformations are quite different, their pairing state paths are highly similar (see Figure S4). As such, this presents an important challenge for analysis by *patteRNA*, which is blind to information on pairing partners and only considers the pairing state of each nucleotide when mining SP data for target structures.

To understand the role of SL III/SL IV in Rev binding, Sherpa et al. isolated two co-existing structural isomers of wild-type HIV-1 pNL4-3 RRE and performed SHAPE. From SHAPE-directed predictions, the authors concluded that each isomer corresponded to the canonical 5SL and 4SL structures [56]. They further produced RRE mutants intended to strengthen or disrupt specific base pairings in the SL III/SL IV region. Their experiments resulted in seven RRE transcripts with SHAPE profiles: two corresponding to the 5SL and 4SL wild-type isomers and five corresponding to mutants denoted A to E. The secondary structures within the SL III/SL IV region (as predicted by Sherpa et al. using data-directed minimum free energy models) of these seven transcripts are shown in Figure 3A–E, with the induced mutations highlighted in red. The binary pairing state paths for each mutant are shown in Figure S4B. Note that Mutants A and B share identical secondary structures with 5SL and 4SL, respectively, as they were designed to stabilize the two wild-type conformations. Moreover, while seven transcripts are considered, the native 4SL and 5SL isomers share the same underlying nucleotide sequence. Hence, this dataset, hereby called the Sherpa set, contains seven SHAPE profiles built from six unique sequences that give rise to five distinct predicted structures. As *patteRNA* represents these structures as pairing-state paths, they are denoted Paths A–E in our subsequent analyses (see Figure S4).

**Figure 4:**
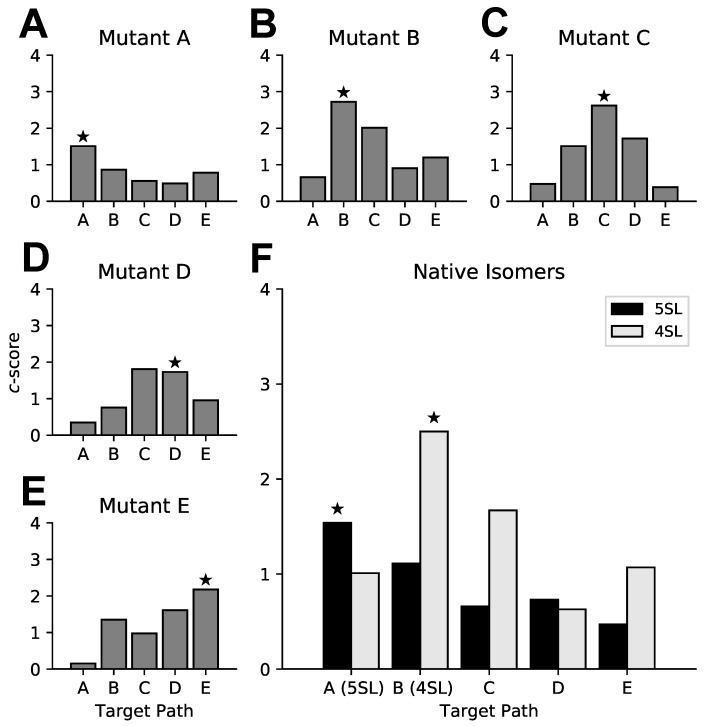
*patteRNA* scores on the Sherpa set of RRE SHAPE (selective 2${}^{\prime }$-hydroxyl acylation analyzed by primer extension) profiles. (**A**–**E**) Each panel corresponds to a SHAPE profile for an RRE mutant. Grey bars indicate *patteRNA*’s *c*-scores for the five Paths A–E. Highlighted with a star is the score for the predicted path in the tested profile. (**F**) *c*-scores for the two native 5SL and 4SL isomers. Bars correspond to scores for Paths A–E on the 5SL (black) and 4SL (grey) profiles. Similar to the other panels, stars highlight scores for the predicted path in each profile, namely Path A for 5SL and Path B for 4SL. All scores correspond to the SL III/SL IV region (nt 163–221).

To determine *patteRNA*’s ability to distinguish between highly similar paths, we searched for each of the structures illustrated in Figure 3 in all seven SHAPE profiles. Note that no sequence constraints were enforced. Our results indicate that, for all but one RRE mutant profile, our algorithm assigns the highest *c*-score to its corresponding predicted path (Figure 4A–E). The exception is Mutant D, where the highest score was given narrowly to Path C over its expected path, Path D (Figure 4D). This misclassification is driven by high reactivities at locations in Path D where nucleotides are expected to be paired (nt 170, 181, 182, and 188). As our algorithm depends solely on data to infer pairing states, Path D is demoted because of direct contradictions between the predicted path and the observed data.

Having demonstrated that *patteRNA* can discriminate between highly similar structural RRE variants, we proceeded to investigate scores for the 5SL and 4SL native isomers. Our results show that the 5SL profile scores highest for Path A, and the 4SL profile scores highest for Path B (Figure 4F). These results are in perfect agreement with Sherpa et al., as Paths A and B correspond to the sequence of pairing states for the predicted 5SL and 4SL native structures, respectively. In summary, our results support the conclusion that the two native isomers are in fact folding into the 5SL and 4SL conformations. Of the two isomers, the 4SL motif appears to be more readily detected by *patteRNA*. This is evidenced by the higher *c*-score when scoring the 4SL profile for its predicted state path, Path B, than when scoring the 5SL profile for its predicted state path, Path A. This difference in *c*-score magnitude indicates that SP data are in stronger agreement with the 4SL isomer predicted structure, compared to the 5SL isomer. This originates from reactivity values in the 5SL profile that contradict the pairing state sequence of Path A. Specifically, nucleotides 169 and 176 are observed to emit high reactivities, despite having been predicted to be in paired states within SL III. Nucleotides 195, 196, 215, 216 comprise an unpaired internal loop within SL IV, however these nucleotides emit very low or zero reactivity. A likely explanation stems from the predicted structure for 5SL having been obtained using a data-driven thermodynamic-based algorithm (RNAstructure) [62]. Such algorithms [29,30,31] consider the possible base-pairing arrangements (not just paired/unpaired states) in the context of the entire RNA and can subsequently consider situations in which a stem-loop is likely to fold, despite the underlying sequence necessitating an internal loop, which may or may not be reactive in SHAPE experiments. As such, it is not surprising to observe deviations between a SHAPE profile and a predicted structure. Given prior knowledge on the structure of RRE, it is possible that the 5SL conformation harbors tertiary interactions altering reactivities at nucleotides in the SL III/SL IV region. We speculate that the 5SL conformation (Path A) could leave the end of stem-loops SL III and SL IV more exposed, subsequently causing heightened reactivities for paired nucleotides. Conversely, low reactivities in the SL IV internal loop may also be explained by the rigidity of the stem-loop. Alternatively, tertiary interactions from other regions of RRE could prevent the internal loop from behaving as unpaired nucleotides in SP experiments.

Overall, these results demonstrate *patteRNA*’s ability to discern structures in SP data, even when trained on relatively small datasets and when tasked with highly similar motifs in terms of their nucleotide pairing states. Although the algorithm’s performance in this situation is not impeccable (i.e., Mutant D is narrowly misclassified as Mutant C), our results are promising given the inherent limitations of our framework, which uses SP data alone and is therefore blind to pairing partners. Scores shown here are specific to the SL III/SL IV region (nt 163–221), however the performance of the algorithm when searching for the full-length versions of Paths A–E convey the same conclusions (Figure S5).

Having observed *patteRNA*’s ability to resolve similar variants of RRE from different SHAPE profiles, we set out to investigate how well it can recognize RRE in the entire HIV-1 genome. At first, this task might seem less challenging in comparison to previous analyses we performed on human transcriptomes [32] due to the relatively small size of the HIV-1 genome. However, the data analyzed in [32] contained mRNAs that are believed to be predominantly unstructured whereas the HIV-1 genome comprises numerous highly structured elements. The latter scenario thus poses a greater challenge in discriminating between signal and background.

We utilized two HIV-1 pNL4-3 SHAPE datasets from the Weeks Lab (Chapel Hill, NC, USA). The first one, by Watts et al. [57], was obtained using the 1M7 reagent and capillary-based cDNA quantification. The second dataset, by Siegfried et al. [47], comprises three SHAPE-MaP profiles probed using 1M6, 1M7 and NMIA reagents. This resulted in a total of four whole-genome profiles, in which we searched for the presence of the five full-length RRE structures (Paths A–E) included in our analysis of the Sherpa set (Figure S3). Note that *patteRNA* training and scoring were performed on each profile independently.

**Figure 5:**
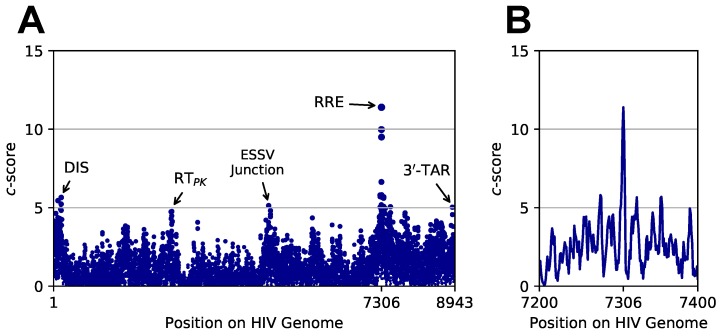
*patteRNA* scores when searching for the 4SL native structure of RRE across human immunodeficiency virus (HIV) genome profiles. (**A**) *c*-scores across the entire HIV-1 RNA genome as probed with *N*-methylisatoic anhydride (NMIA) by Siegfried et al. The peak at nucleotide 7306 corresponds to the known start site of the RRE. Other labeled peaks correspond to known structured elements in HIV-1. Scores end at nucleotide 8943 as this is the last location in the 9174-nt genome able to accept the 232-nt target paths. (**B**) Inset of *c*-scores around the RRE start site.

Our results show that our algorithm successfully identified the exact location of the RRE structure in all four profiles (Figure 5, see Figure S6 for complete scoring results). This is demonstrated by highest *c*-scores falling precisely at the expected start location of RRE (nucleotide 7306, Table 1). Table 1 contains the highest scoring site in the whole-genome profiles for each of the five paths, A–E. Interestingly, top scores at this site are given to either the 4SL or 5SL native structures in all profiles. This is expected, as Paths C–E correspond to RRE mutants whose mutations were created artificially to render native conformations unfeasible. Note, however, that Paths C–E are still detected because we searched for the full-length RRE motif, while induced mutations are understood to drive structural rearrangements only within the SL III/SL IV region. In other words, all targets have identical structures outside of SL III/SL IV, meaning that differences in scores primarily relate to reactivity differences in only 59 out of the 232 nucleotides in RRE.

While the true site of RRE is consistently assigned the highest *c*-score over all sites in each genome, we also observed signals at other structured regions of HIV. For example, the dimerization initiation site (DIS), reverse transcriptase pseudo-knot (RT${}_{PK}$), exonic splicing silencer ESSV junction, and 3${}^{\prime }$-TAR all give rise to detectable *c*-score peaks (Figure 5A). Because the searched RRE motif is highly structured (>65% paired states), it is not surprising to observe heightened scores at other highly structured regions. Interestingly, the structure of the ESSV junction is not well characterized, however, recent studies have identified this region as structurally conserved across HIV and simian immunodeficiency virus (SIV) [64]. Our observations suggest that this region, readily known to influence transcription and replication [78,79], may harbor an intricate structure related to its roles in splicing.

Large fluctuations in *c*-scores were also observed in the vicinity of the known location of RRE (Figure 5B). These are due to pairing state agreements and contradictions when sliding the target motif’s path around the true site of RRE. Because RRE is comprised by stretches of paired and unpaired nucleotides, the overlap between pairing states of the target path and those of the underlying structure of RRE will vary greatly as the target path is considered near the true site. Finally, we observe that the 4SL structure consistently ranks as the top scorer, indicating that it may be the dominant conformation in the HIV-1 genomes probed in these studies.

In addition, to place these results in the context of searches in larger datasets, we conducted a search for the two native conformations of the full-length RRE (5SL/Path A and 4SL/Path B) in a subset of highly data-dense transcripts from a human transcriptome-wide PARS dataset [37]. Searches were conducted without sequence constraints. To establish the theoretical rank that the RRE would be assigned if present in human data, *c*-scores obtained at the location of the RRE in all HIV-1 SHAPE profiles (see Table 1 for details) were ranked against the *c*-scores from the PARS searches for both 5SL and 4SL. Our results indicate that both conformations would rank first out of 1,114,957 sites in the PARS dataset for all HIV-1 genomes (see Figure S7). This suggests that the RRE would be easily identified even at a scale much larger than a 9kb viral genome. In addition, note that the distribution of *c*-scores is skewed towards high values for searches in the HIV-1 genome, as viral genomes are understood to be generally much more structured compared to mRNAs. Such statistical differences underscore the additional challenge faced by searches in relatively structured domains.

Having observed that *patteRNA* can find the RRE motif in whole-genome SHAPE profiles, we performed a similar search, but instead, we considered the SL III/SL IV region exclusively. This search provides a more specific measure of the structural nature of SL III/SL IV without overwhelming signal from the rest of the full-length RRE. The results of this search (Table S1) are in line with our full-length search results. Depending on the reagent used, *c*-scores indicate the either the 4SL conformation is dominating (Siegfried set, NMIA and 1M7) or both the 5SL and 4SL conformations co-exist (Siegfried set, 1M6; Watts set, 1M7). When searching the smaller 59-nt motif, less information on the target results in a reduced signal at the true positive site of SL III/SL IV (nt 7409–7467) compared to the rest of the genome. Subsequently, this site is not assigned the highest *c*-score across all sites. As such, we include in Table S1 the percentile of *c*-scores to highlight the ranking of the site relative to the rest of the data. We also repeated this search with sequence constraints enforced, to prune scores corresponding to sites with nucleotide sequence precluding formation of the target path. Despite the shorter length of the SL III/SL IV target paths, the true site of RRE is nevertheless the only site in the data satisfying sequence constraints. Paths A, B, and E satisfy sequence constraints at this site only, while Paths C and D are rendered invalid because of base pairings deriving from induced mutations. Moreover, Path E, which satisfies sequence constraints at the true site in the data, is assigned a relatively low *c*-score compared to the native isomers 5SL/4SL. Taken together, these results support previous work concluding that 5SL and 4SL are the true underlying structures of RRE.

Having established that *patteRNA* distinguishes between 5SL and 4SL structures, we investigated its ability to resolve them from profiles of heterogeneous samples where they co-exist. Sherpa et al. concluded that RRE could exist as a mixture of these two structures and demonstrated that they are not functionally equivalent. More generally, the ability of structural elements to assume more than one conformation is often critical for regulatory flexibility and sensitivity. Detecting changes in the relative abundances of alternative structures is therefore an important, yet challenging problem in biology.

To explore *patteRNA* scoring of ensembles of RRE, we simulated SHAPE profiles for mixtures of the 5SL and 4SL isomers ranging from 100% 5SL to 100% 4SL, in 10% increments. Each mixture was generated by summing the desired weight fraction of the Sherpa set profiles at each nucleotide.Because we generated mixtures from the pure isomer profiles, these data essentially emulate SHAPE profiles for ensembles comprised of varying proportions of 5SL/4SL structures. Mixtures were thenscored against the 5SL and 4SL motifs, similar to the analysis performed in Figure 4. Here, results are considered as *c*-score ratios between the 5SL and 4SL targets ($c_{5\mathrm{SL}}/c_{4\mathrm{SL}}$). This ratio is indicative of the relative likelihood of the two targets given their respective *c*-scores. Starting with 100% 5SL (Figure S8), our results reveal that scores evolve monotonically from favoring 5SL until 30% of the profile is comprised of the 4SL SHAPE data, at which point scores favor 4SL, as indicated by ratios below 1. This demonstrates that *c*-scores reflect the gradient of mixture composition underlying the simulated data.

Although *patteRNA* was not developed to decipher ensemble dynamics, our results suggest that it can readily detect composition changes in simulated data. This also hints at further applications to statistically quantify changes in structural ensembles over a time series or differing experimental conditions. Importantly, while our results suggest *patteRNA* could be utilized as a tool to detect relative changes in ensemble composition, the exact estimation of underlying population fractions remains a challenge currently beyond the algorithm’s capabilities. We therefore recommend the use of other data-directed methods designed to determine ensemble compositions when approaching this problem [59,60,61]. Nevertheless, the utility of *patteRNA* in differential analyses of ensemble composition is promising.

In summary, we have demonstrated that *patteRNA* is capable of discerning subtle structural variation directly from SHAPE data. At the same time, it can also detect structural motifs in the larger context of a whole viral genome. Beyond basic structure characterization and search, we have shown that *patteRNA* can glean quantitative insights on changes in ensemble compositions of native RRE isomers. Notably, all results were obtained using a standard laptop (Intel 3.1 GHz i7 CPU and 16 GB of RAM) and completed in just a few minutes, even for whole-genome HIV datasets.

### 3.4. Automating patteRNA’s Training Routine

Expectation-Maximization training algorithms sometimes suffer from slow convergence. To reduce runtime, a trivial solution is to reduce the size of the training set to its bare minimum. We previously noted that *patteRNA* can be trained on a subset of the data as long as it is statistically representative of the full dataset [32]. However, we did not provide specific guidelines regarding the exact number of data points, or transcripts, to be used for training, as those could vary widely based on the probing technique, sequencing approach, and data quality [80]. To circumvent this issue, we implemented an automated procedure to build the training set based on a KL divergence criterion. Briefly, transcripts are added sequentially to the training subset and the KL divergence computed. We stop adding transcripts when the reactivity distribution of the training set is sufficiently close to the distribution of the entire dataset, as indicated by a small KL divergence. The resulting training set is generally much smaller compared to the entire dataset, thereby reducing computational requirements considerably, while arriving at a trained model still representative of the whole data as demonstrated in Figure S9.

Next, a central parameter in *patteRNA* is the number of Gaussian components (*K*) used by the GMM to link reactivities to pairing states. *K* controls the smoothness of the model and if *K* is too small for the considered data, then the model will not capture all the statistical characteristics of the data, thereby leading to prediction inaccuracies. On the other hand, as *K* increases, the model requires more computational resources, both in runtime and in memory. In the original implementation of the algorithm, *K* was a user-defined parameter, as an optimal *K* depends on the considered data and can thus vary greatly. However, determining an optimal *K* is difficult as a grid-like search over selected *K* values is required, followed by a manual inspection of the model’s goodness-of-fit. To alleviate these issues, we implemented an automated detection of *K* which utilizes BIC to select among models with increasing *K* (see Materials and Methods).

Finally, we implemented the option to initialize the model in a supervised manner. Briefly, if reference structures are provided, we utilize them to find the best estimates of the initial parameters of the GMM-HMM model. In the more common scenario of unsupervised training, we modified parameter initialization so that both the GMM and HMM part of *patteRNA* are more representative of a biologically meaningful solution. For this, we use initial HMM parameters derived from the Weeks set that we described previously [32]. For the GMM, we use the percentiles of the reactivity distribution to gauge the initial location (i.e., the mean) of the Gaussian components (see Section 2.2.3 in Materials and Methods).

## 4. Discussion

The emergence of high-throughput SP experiments offers new opportunities to study the functional roles of RNA structures at the transcriptome level and in both in vitro and in vivo conditions. This new wealth of data has given rise to a critical need for methods that facilitate rapid data-driven inference of structures in large datasets. *patteRNA* is a first step toward closing this gap. We envision our algorithm’s utility to be twofold. First, it provides a novel approach to identifying RNA elements, which scales well with both RNA length and the number of analyzed transcripts. This means that any functional RNA element with a known or predicted secondary structure can be mined and studied quantitatively in the context of the entire transcriptome. Specific applications include identifying de novo sites harboring a functional motif (for example, identifying regulatory elements such as splice sites, riboswitches, or thermosensors), quantifying that motif’s prevalence globally or its enrichment in defined RNA regions (e.g., 5${}^{\prime }$-UTR or 3${}^{\prime }$-UTR), and quantitatively studying the impact of varying experimental conditions on the presence of said motif. When considered in the context of other structure analysis tools, our algorithm could be useful in maximizing insights derived from these tools. Specifically, for large datasets, *patteRNA* can be used to select a small list of candidate regions that can then undergo more careful characterization with targeted probing, thermodynamic modeling, or phylogenetic analysis. Additionally, while we believe these capabilities are valuable for basic research, they may prove useful for the design of RNA-based therapeutics [81,82,83] by identifying putative target and off-target sites of drugs designed to bind RNA or by identifying RNA designs that bind a target molecule. Second, our probabilistic framework can be applied universally across SP methods and probes, bridging their differences and standardizing the interpretation of SP data as probability estimates of nucleotide pairing states. Importantly, we do not foresee *patteRNA* replacing existing methods for secondary structure prediction [29,30,31] or for functional RNA element mining from homologous sequences. We rather intend it to complement these tools. Of particular relevance are covariance models, which are built from a stochastic context-free grammar framework trained on phylogenetic sequence information [84,85]. Previously trained covariance models of RNA families can additionally be utilized to search for similar structures within genomic datasets. This approach is analogous to *patteRNA*, in that a statistical model is trained and subsequently utilized to search and score possible matches in large datasets. While *patteRNA* aims to capture structural information from SP data alone, covariance models aim to capture it from just nucleotide sequence. As such, these approaches capture structural information at distinct levels. The prospect of a unified probabilistic framework capturing information from both sequence and probing data therefore presents an intriguing challenge for the field.

A central requirement for comparing results across distinct structural motifs and datasets is the assurance that scores convey the same information about the presence or absence of a target motif at a site, regardless of the chosen target and dataset. A robust training routine is the first means by which to ensure fair comparison of results between datasets. This can be achieved by defining strict procedures on the determination of initial model parameters that require no user inputs and promote convergence of the model to biologically relevant solutions. To this end, we implemented several improvements and routines that fully automate *patteRNA*, enhancing its ease-of-use and training robustness. We next aimed to assure that inherent properties of the target motif would not bias results. In practice, this requirement was not always met, as scores displayed dependencies on motif length and paired/unpaired composition. Moreover, the proportion of paired/unpaired nucleotides in the considered dataset might also impact scores, furthering the discrepancies between searches. Put simply, scores might be incomparable between distinct searches. To alleviate this issue, we implemented a normalization routine that converts raw scores into *c*-scores. To this end, we used sequence information to classify scored sites as either putative positives or not harboring the target motif (null sites). Scores at null sites are then used to build a null distribution of scores, against which we referenced scores for putative positive sites. Intuitively speaking, a *c*-score is simply the −$\log_{10}$ of a *p*-value, thereby converting raw scores to a generalized measure of significance. While our strategy remedied the biases inherent to searching different motifs, it should be noted that the presence of missing reactivities in the data might nevertheless lead to additional biases. This arises from the bias inherent to motif length. Specifically, missing values are treated as “no information” and hence marginally contribute to raw scores. Scoring a region with sparse data is thus analogous to scoring a shorter motif. Consequently, sites containing missing reactivities tend to span a narrower range of scores, compared to sites with complete observations. This in turn reduces their likelihood to emerge as top candidates in searches. This issue should be kept in mind when using *patteRNA* to search sparse datasets or poorly covered transcripts [80]. Furthermore, note that this bias cannot be easily corrected for as this necessitates considering all possible missing data patterns, a combinatorial problem that is computationally prohibitive.

Another consideration when mining large datasets for a motif is the vast number of negative sites which can often lead to signal depletion and obfuscate correct classification of true positives. As such, the pronounced signal for RRE detected by *patteRNA* is promising even in the context transcriptome-wide searches. It should be noted that sequence constraints can significantly enhance accuracy and precision during these searches because a significant number of negative sites will be pruned, henceforth enriching the scored data for true positives. This filtering is often helpful in the context of high-throughput SP data, where the overwhelming majority of sites are expected to be true negatives. However, sequence constraints might not always be relevant.

This mainly stems from *patteRNA*’s broad applicability to search target structures that are not necessarily nested, or may not be captured by the traditional prediction paradigm of secondary structure. For example, a data signature could capture not only canonical base-pairing interactions but also inter-/intra-molecular interactions [86]. In such cases, the sequence of pairing states in the target would be representative of unreactive/reactive nucleotides rather than paired/unpaired ones. Additional situations where sequence constraints are not applicable include searches for regions that are highly accessible (e.g., loops) [87] or highly structured [47].

Finally, we considered several conformations of the RRE element in HIV-1 to assess the discriminatory power of our revised pipeline. RRE is essential for viral replication and its structure has been extensively studied, providing a well-characterized element by which to benchmark our algorithm. We showed that *patteRNA* successfully identifies RRE structure variants—a non-trivial task considering the high similarity between their pairing state sequences (Figure 3, Figure S4). These results also indicate that high-quality SHAPE data alone could suffice to resolve alternative target motifs at a site, even when the targets share many similarities. We then used simulations to demonstrate the capability to discern changes in ensemble composition as such analyses do not directly depend on a precise determination of the proportion of each underlying conformation. Next, we searched for RRE across whole HIV-1 profiled genomes and demonstrated that *patteRNA* easily and consistently finds its known location across four independent SHAPE profiles. Importantly, different SHAPE reagents were used and analyzed separately, thereby highlighting the algorithm’s robustness. Of note is the remarkable signal-to-noise ratio between the location of the RRE compared to the rest of the HIV genome. This is likely because the RRE is quite large (232-nt), such that sufficient conclusive information can be gleaned to confidently discriminate between its presence and absence. Interestingly, we detected additional signals at other well-characterized and highly structured HIV-1 elements, such as the DIS, reverse-transcriptase pseudoknot (RT${}_{PK}$), and 5${}^{\prime }$-TAR. Moreover, our search revealed a highly structured context around the exonic splicing silencer ESSV, which to our knowledge has not previously been subjected to targeted structure probing outside of whole-genome studies. Taken together, our results highlight that future application of data-driven methods to other RNA viruses [88,89] and whole-transcriptomes has the potential to detect novel structured elements and changes to them.

## Figures and Tables

**Table 1:** Highest *patteRNA* scores when searching Rev response element (RRE) motifs across four whole-genome human immunodeficiency virus type 1 (HIV-1) SHAPE (selective 2${}^{\prime }$-hydroxyl acylation analyzed by primer extension) profiles. Genomes were searched for the five RRE structures reported in the Sherpa set. All top *c*-scores occur at the known site of RRE in the HIV-1 pNL4-3 genome (nt 7306–7537).

Dataset	Reagent	Search Target	Top *c*-Score
		Path A (5SL)	10.6
		Path B (4SL)	11.4
	NMIA	Path C	11.0
		Path D	10.6
		Path E	11.4
		Path A (5SL)	12.2
		Path B (4SL)	12.4
Siegfried Set	1M6	Path C	11.5
		Path D	12.4
		Path E	12.4
		Path A (5SL)	11.5
		Path B (4SL)	13.0
	1M7	Path C	12.2
		Path D	11.8
		Path E	11.9
		Path A (5SL)	12.9
		Path B (4SL)	13.2
Watts Set	1M7	Path C	12.7
		Path D	11.9
		Path E	12.6

NMIA: *N*-methylisatoic anhydride; 1M6: 1-methyl-6-nitroisatoic anhydride; 1M7: 1-methyl-7-nitroisatoic anhydride.

## Supplementary Materials

The following are available online at http://www.mdpi.com/2073-4425/9/6/300/s1, Figure S1: Initialization of four Gaussian components using data percentiles, Figure S2: Illustration of sequence constraints, Figure S3: Secondary structures of the in vitro RREs, Figure S4: Sequences and pairing state paths of the SL III/SL IV region for RRE variants in the Sherpa set, Figure S5: *patteRNA* scores on the Sherpa set of RRE SHAPE profiles when searching full-length RRE paths, Figure S6: *patteRNA* scores for RRE motifs across four whole-genome HIV-1 structure profiles, Figure S7: Survival functions of *c*-scores for the 5SL and 4SL native structure of RRE across human transcriptome-wide PARS and HIV-1 SHAPE datasets, Figure S8: *patteRNA* score ratios (5SL/4SL) for mixtures of the 5SL and 4SL native isomers of the RRE, Figure S9: Comparison of trained models using an entire dataset and a reduced training subset, Table S1: *patteRNA* scoring of the SL III/SL IV region (nt 7409–7467) of RRE in genomic SHAPE data against the candidate paths described in the Sherpa set. *patteRNA* is written in Python3, is multi-platform, and is multi-core enabled. The source code is freely available on GitHub at https://github.com/AviranLab/patteRNA under the BSD-2 license (release v1.2.0). Data and analysis scripts supporting the conclusions of this article are freely available at https://doi.org/10.5281/zenodo.1256866 [90].

## Appendix A

Initial parameters of *patteRNA*’s Gaussian Mixture Model-Hidden Markov Model (GMM-HMM) when no reference structures are provided, i.e., under unsupervised initialization:

- Number of Gaussian components per pairing state (*K*): Auto-detected using Bayesian Information Criterion (BIC)
- Transition probabilities (derived from the Weeks set):
  $$\left[\begin{array}{ccc} & \mathrm{Unpaired} & \mathrm{Paired}\\ \mathrm{Unpaired} & 0.71020019 & 0.28979981\\ \mathrm{Paired} & 0.19677996 & 0.80322004 \end{array}\right]$$
- Initial probability:
  $$\left[\begin{array}{cc} \mathrm{Unpaired} & \mathrm{Paired}\\ 0.5 & 0.5 \end{array}\right]$$
- Gaussian means: Based on data percentiles
- Gaussian variances: Equal to the variance of the data
- Gaussian weights: $\frac{1}{K}$
