# Supplementary material for: Automated Recognition of RNA Structure Motifs by Their SHAPE Data Signatures
Source: Genes (Basel). 2018 Jun 14;9(6):300. doi: 10.3390/genes9060300 (PMC6027059; doi:10.3390/genes9060300)
Supplement: Supplementary file 1 [file genes-09-00300-s001.pdf]

Supplementary Materials: Automated recognition of RNA structure motifs by their SHAPE data signatures

Pierce Radecki, Mirko Ledda, and Sharon Aviran

- Figure S1: Initialization of four Gaussian components using data percentiles.
- Figure S2: Illustration of sequence constraints.
- Figure S3: Secondary structures of the *in vitro* RREs.
- Figure S4: Sequences and pairing state paths of the SL III/SL IV region for RRE variants in the Sherpa set.
- Figure S5: *patteRNA* scores on the Sherpa set of RRE SHAPE profiles when searching full-length RRE paths.
- Figure S6: *patteRNA* scores for RRE motifs across four whole-genome HIV-1 structure profiles.
- Figure S7: Survival functions of *c*-scores for the 5SL and 4SL native structure of RRE across human transcriptome-wide PARS and HIV-1 SHAPE datasets.
- Figure S8: *patteRNA* score ratios (5SL/4SL) for mixtures of the 5SL and 4SL native isomers of the RRE.
- Figure S9: Comparison of trained models using an entire dataset and a reduced training subset.
- Table S1: *patteRNA* scoring of the SL III/SL IV region (nt 7409-7467) of RRE in genomic SHAPE data against the candidate paths described in the Sherpa set.

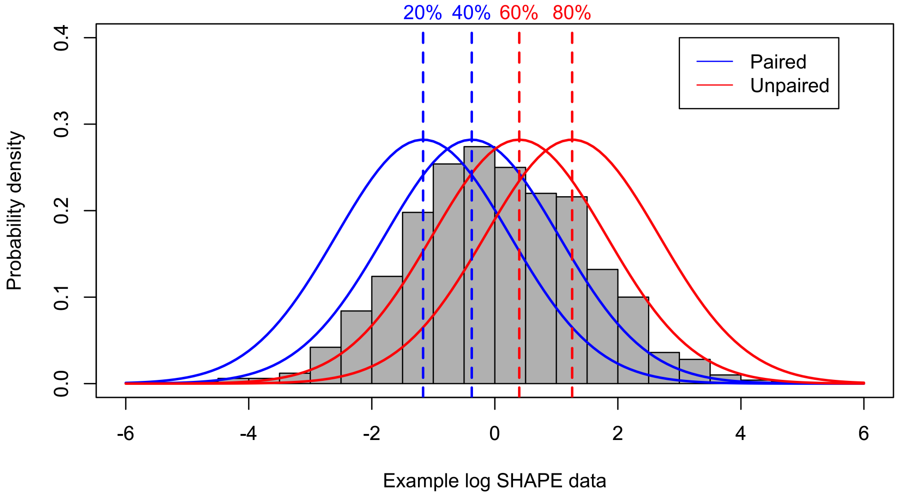

Figure S1. Initialization of four Gaussian components using data percentiles. Grey histograms represent the distribution of example data. In this case, the parameter $K = 2$ (i.e., two components per pairing state) and each Gaussian component is represented by a solid line with blue indicating the two components used to model paired nucleotides, and red, unpaired ones. Gaussian means are spaced at regular percentile intervals, in this case at 20%, 40%, 60% and 80% of the data distribution density, respectively.

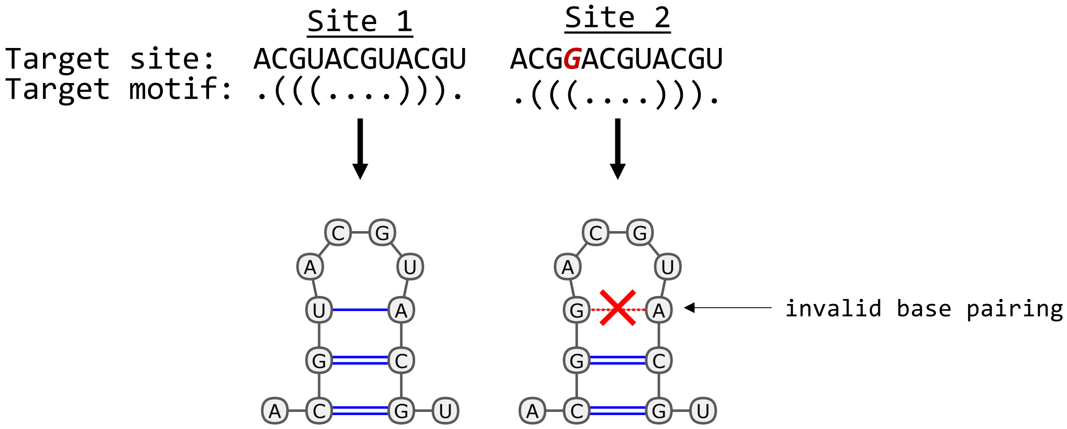

Figure S2. Illustration of sequence constraints. When comparing the target motif to the nucleotide sequence in Site 1, all base pairings follow the canonical rules (G–C, A–U, G–U allowed). This site consequently "passes" sequence constraints. On the contrary, the nucleotide sequence in Site 2 gives rise to non-canonical base pairings. Specifically, a G–A pairing is deemed invalid. As such, this site violates sequence constraints.

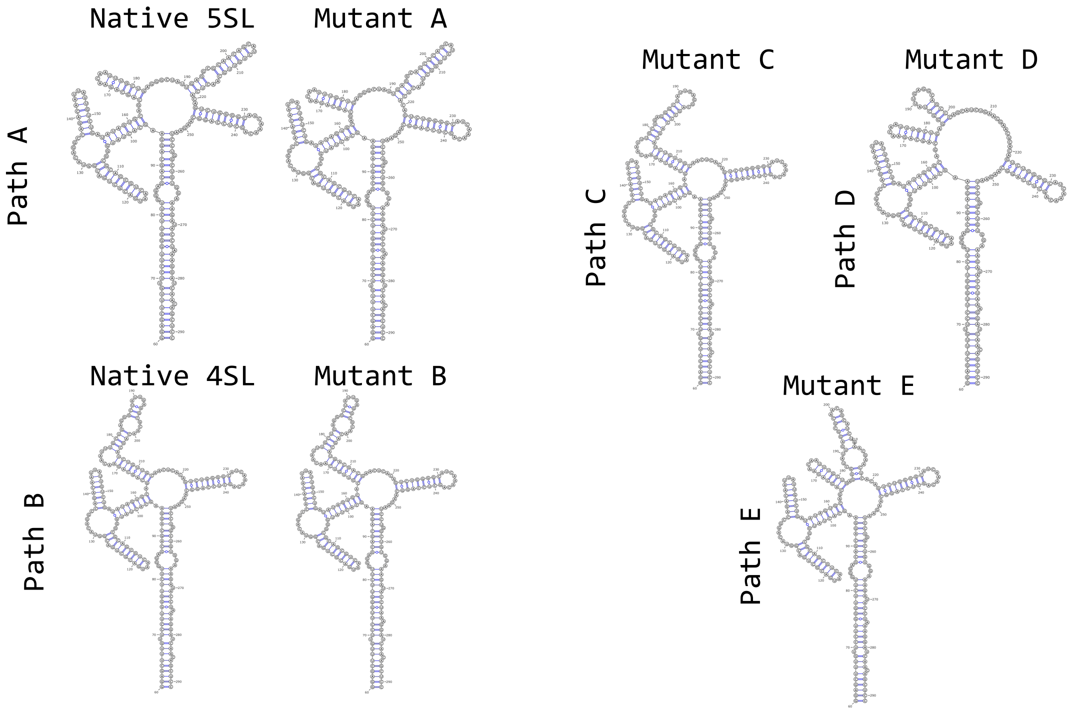

Figure S3. Secondary structures of the *in vitro* RREs (nt 60-291), as predicted by Sherpa *et al*.

A

```
            nt. 163
              ↓    SL III region              SL IV region
        5SL  AGCAGCAGAACAAUUUGCUGAGGGCUAUUGAGGCGCAACAGCAUCUGUUGCAACUCACA
        4SL  AGCAGCAGAACAAUUUGCUGAGGGCUAUUGAGGCGCAACAGCAUCUGUUGCAACUCACA
   Mutant A  AGCAGCAGAACAAUUUGCUGAGGGCUAUUGAGGCAGCCCAGCAUCUGGGCUAACUCACA
   Mutant B  ACGUUGUCAACAAUUUGCUGAGGGCUAUUGAGGCGCAACAGCAUGACGACGAACUCACA
   Mutant C  AGCAGCAGAACAAUUUGCUGAGGGCUAUUGAGGCAGCCCAGCAUCUGUUGCAACUCACA
   Mutant D  AGCAGCAGAACAAUUUGCUGAGGGCUAUUGAGGCAGCCCAGCAUCUGAACGAACUCACA
   Mutant E  AGCAGCAGAACAAUUUGCUGAGGGCUAUUGAGGCGCAACAGCAUCUGAACGAACUCACA
```

B

```
              nt. 163
                ↓
5SL/Mutant A  00111111100001111111000000001111001111111000111111100111100  ← Path A
4SL/Mutant B  01111111100000011111000111000001110000111111111111100000000  ← Path B
    Mutant C  01111111100000011110011111000000001111111111111111100000000  ← Path C
    Mutant D  00111111100001111111011111000000001111100000000000000000000  ← Path D
    Mutant E  00111111100001111111011100001011101100001101110100000111000  ← Path E
```

Figure S4. Sequences and pairing state paths of the SL III/SL IV region for RRE variants in the Sherpa set. **(A)**: Nucleotide sequences for the SL III/SL IV region (nt 163-221) in RRE included in the Sherpa set. In the 5SL structure, SL III and SL IV fold into distinct stem-loops (indicated in grey). In the 4SL structure, these two stem-loops rearrange and merge to form a single larger stem-loop known as SL III/IV. Mutations are highlighted in yellow with bold text. **(B)**: Binary pairing state representation of the native isomers and mutants of RRE within SL III/SL IV. Unpaired and paired nucleotides are represented by 0 and 1, respectively. Secondary structures related to these sequences are illustrated in Figure S3.

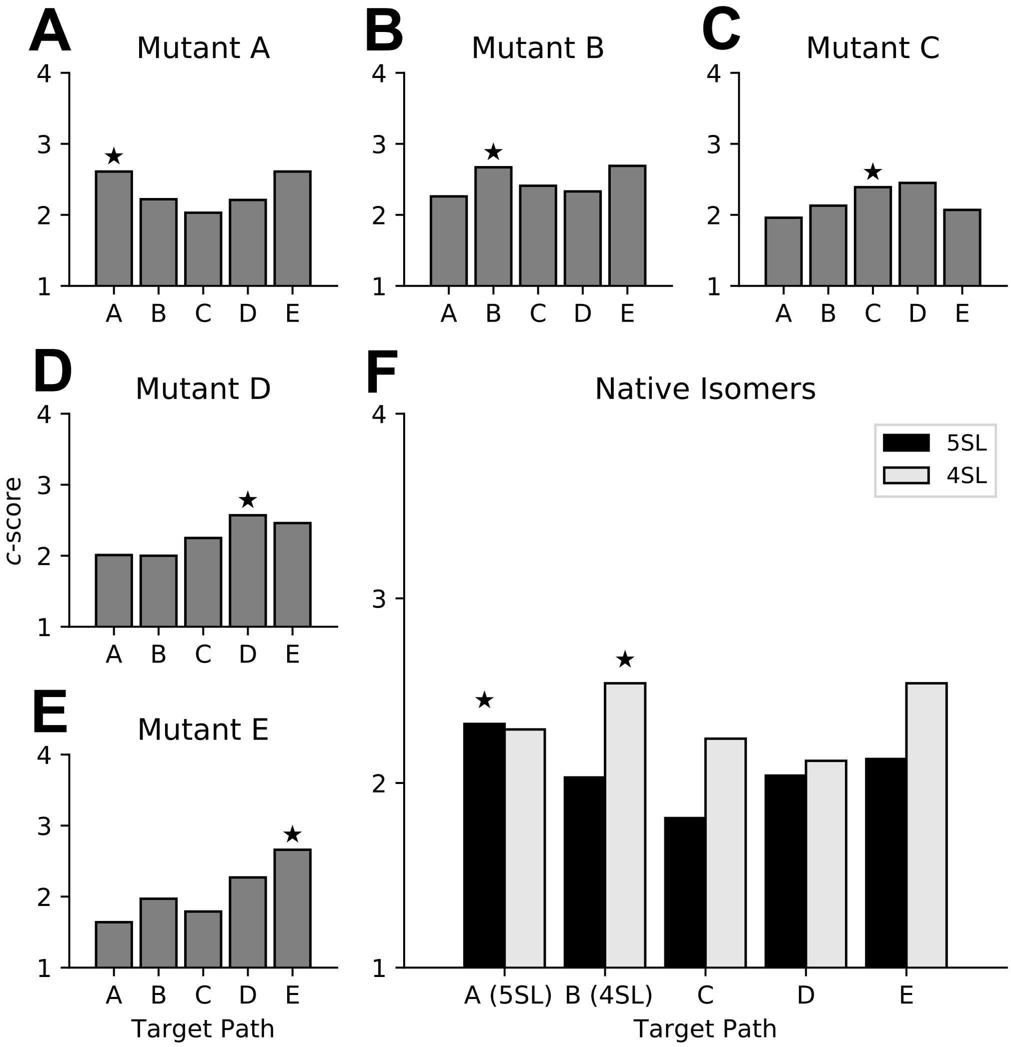

Figure S5. *patteRNA* scores on the Sherpa set of RRE SHAPE profiles when searching full-length RRE paths. **(A-E)** Each panel corresponds to a SHAPE profile for an RRE mutant. Grey bars indicate *patteRNA*'s *c*-scores for the five paths A-E. Highlighted with a star is the score for the predicted path in the tested profile. **(F)** *c*-scores for the two native 5SL and 4SL isomers. Bars correspond to scores for paths A-E on the 5SL (black) and 4SL (grey) profiles. Similar to the other panels, stars highlight scores for the predicted path in each profile, namely path A for 5SL and path B for 4SL. Note that *y*-axes start at 1 to better highlight differences in *c*-scores between paths, which relate primarily to differences in 59 out of 232 nucleotides when searching the full-length path.

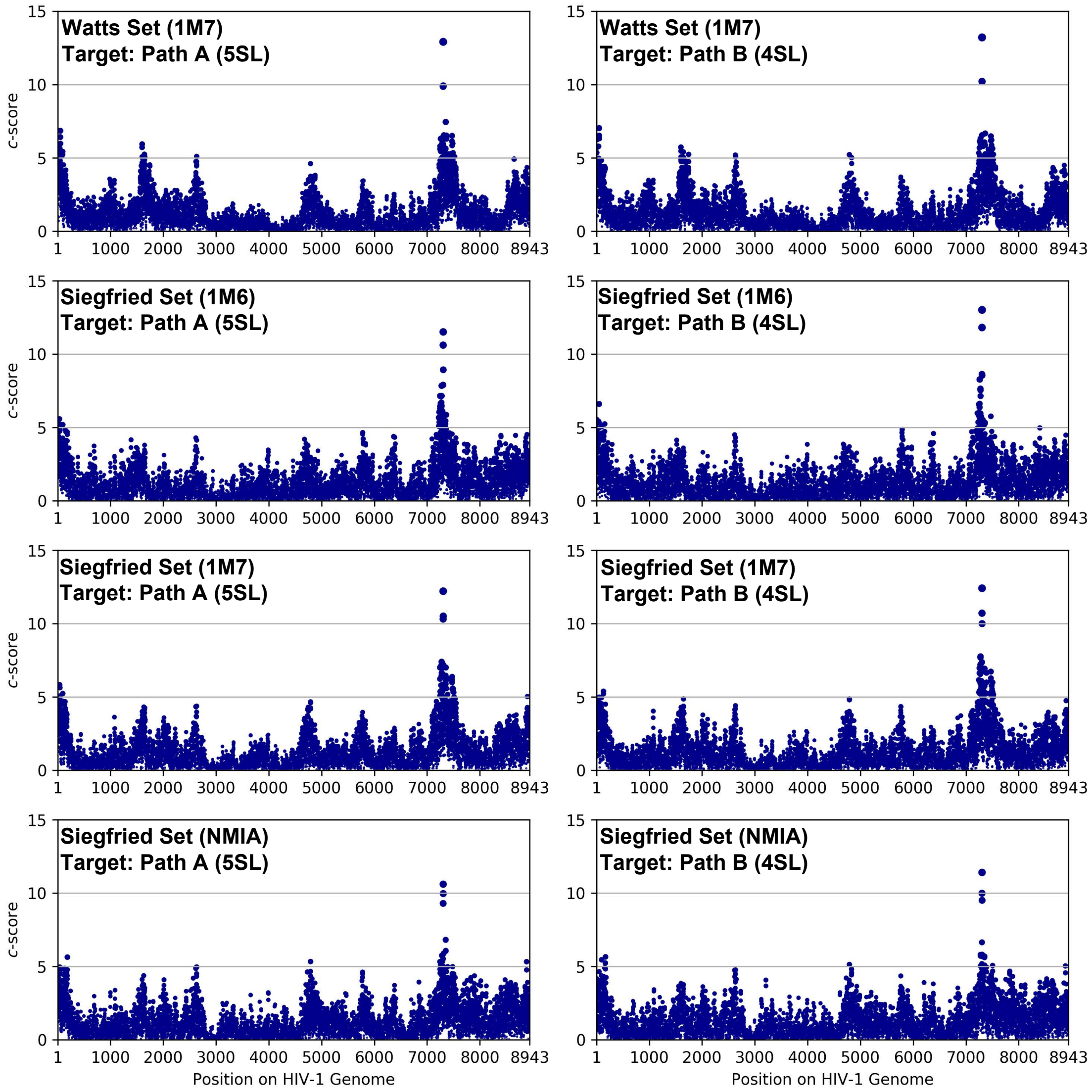

Figure S6. *patteRNA* scores for RRE motifs across four whole-genome HIV-1 structure profiles. *c*-scores for full-length paths A (5SL structure, left panels) and B (4SL structure, right panels) across all sites in the HIV-1 genome. Dataset and modifying reagents used are indicated in each panel and include the Watts set (SHAPE assayed with 1M7) and three profiles from the Siegfried set (SHAPE-MaP assayed with 1M6, 1M7, and NMIA, respectively). Peaks at nucleotide 7306 correspond to the known start location of the RRE.

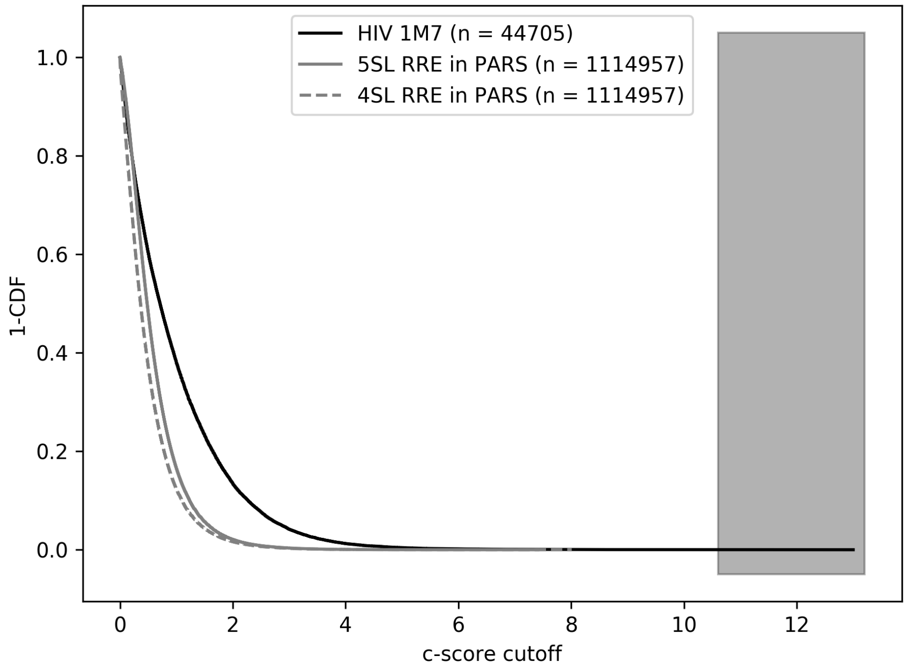

Figure S7. Survival functions of c-scores for the 5SL and 4SL native structure of RRE across human transcriptome-wide PARS and HIV1 SHAPE datasets. We report c-scores for searches conducted across 649 transcripts in the PARS set with data density above 75% (i.e. $\leq$ 25% missing data), as well as c-scores from the entire HIV-1 RNA genome as probed with 1M7 by Siegfried *et al.*. The y-axis represents the proportion of data points with c-scores above the cutoff reported on the x-axis, i.e. the survival function defined as $1 - \mathrm{CDF}(c)$, where $\mathrm{CDF}(c)$ is the cumulative distribution function. The grey rectangle highlights the dynamic range of c-scores (10.6 to 13.2) obtained at the location of the RRE for all considered RRE paths and HIV-1 SHAPE profiles (see Table 1 for details).

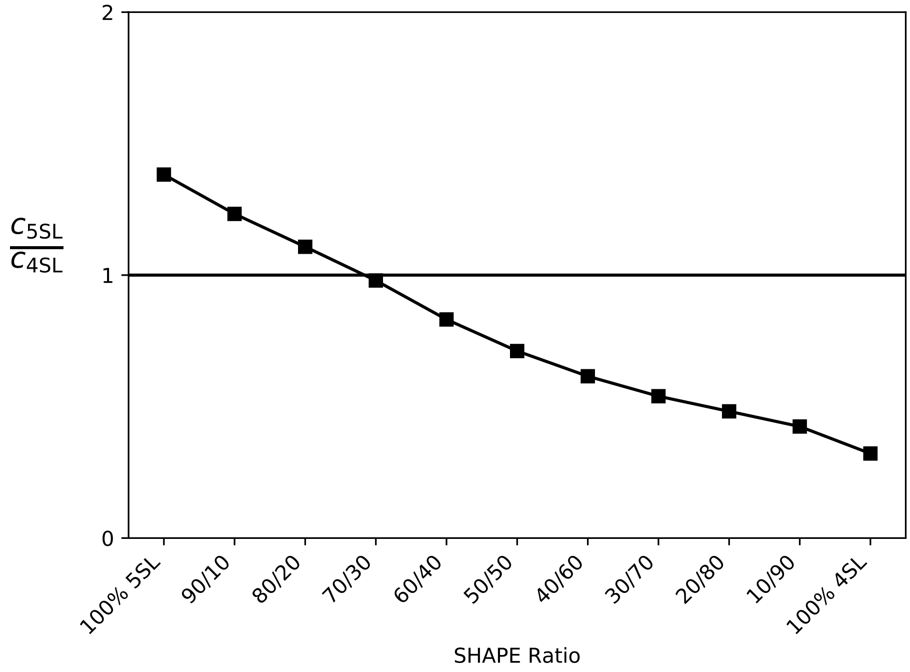

Figure S8. *patteRNA* score ratios (5SL/4SL) for mixtures of the 5SL and 4SL native isomers of the RRE. The x-axis corresponds to SHAPE profiles emulating various mixtures of the 5SL/4SL conformations. The y-axis corresponds to c-score ratios between the 5SL and the 4SL paths (c_{5SL}/c_{4SL}). Results indicate a stable progression of -score ratios initially favoring the 5SL structure until the SHAPE data is comprised by 30% 4SL, at which point the 4SL structure receives higher scores.

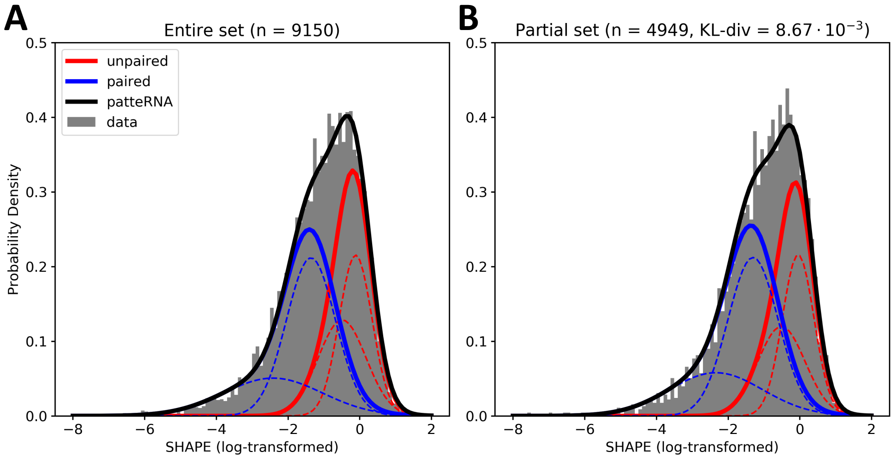

(A) Optimal K: 2

Initial probabilities:

Unpaired	Paired
0.41	0.59

Transition probabilities:

	Unpaired	Paired
Unpaired	0.83	0.17
Paired	0.13	0.87

State probabilities:

Unpaired	Paired
0.41	0.59

(B) Optimal K: 2

Initial probabilities:

Unpaired	Paired
0.36	0.64

Transition probabilities:

	Unpaired	Paired
Unpaired	0.83	0.17
Paired	0.12	0.88

State probabilities:

Unpaired	Paired
0.44	0.56

Figure S9. Comparison of trained models using an entire dataset and a reduced training subset. The input data are based on the HIV-1 genome probed with 1M7 from the Siegfried set and partitioned into 100 bp fragments to mimic multiple transcripts. Gaussian Mixture Models (black lines) learned by *patteRNA* as well as Hidden Markov Model parameters for **(A)** the entire dataset and **(B)** a training subset determined using KL-divergence. Grey histograms represent the distribution of the SHAPE data. Distributions associated with paired and unpaired nucleotides are shown in blue and red solid lines, respectively(solid colored lines). Individual Gaussian components are highlighted by dashed colored lines (two for each pairing state as the optimal $K = 2$ for this dataset).

Table S1. *patteRNA* scoring of the SL III/SL IV RRE region (nt. 7409-7467) in genomic SHAPE data against the candidate paths A–E described in the Sherpa set. *c*-scores with and without sequence constraints are included. Paths C and D violate sequence constraints and are not reported. *c*-score percentiles are included to gauge the significance of the reported values. Percentiles correspond to the percentage of scored sites falling below the reported *c*-score.

Dataset	Reagent	Search Target	*c*-score (Percentile) *no sequence constraints*	*c*-score *sequence constraints*
Siegfried Set	NMIA	5SL/Path A	0.75 (81st)	0.75
		4SL/Path B	1.58 (97th)	1.52
		Path C	1.18 (93rd)	*invalid*
		Path D	0.23 (41st)	*invalid*
		Path E	0.83 (84th)	0.78
	1M6	5SL/Path A	1.39 (95th)	1.39
		4SL/Path B	1.22 (94th)	1.22
		Path C	0.55 (71st)	*invalid*
		Path D	0.50 (67th)	*invalid*
		Path E	0.65 (77th)	0.65
	1M7	5SL/Path A	1.42 (96th)	1.48
		4SL/Path B	2.77 (99th)	2.75
		Path C	2.02 (99th)	*invalid*
		Path D	0.81 (84th)	*invalid*
		Path E	0.98 (89th)	0.99
Watts Set	1M7	5SL/Path A	2.60 (99th)	2.60
		4SL/Path B	2.50 (99th)	2.50
		Path C	2.18 (99th)	*invalid*
		Path D	0.16 (31st)	*invalid*
		Path E	0.45 (63rd)	0.45
